# Polarization Controllable Device for Simultaneous Generation of Surface Plasmon Polariton Bessel-Like Beams and Bottle Beams

**DOI:** 10.3390/nano8120975

**Published:** 2018-11-26

**Authors:** Peizhen Qiu, Taiguo Lv, Yupei Zhang, Binbin Yu, Jiqing Lian, Ming Jing, Dawei Zhang

**Affiliations:** 1 Engineering Research Center of Optical Instrument and System, Ministry of Education and Shanghai Key Laboratory of Modern Optical System, University of Shanghai for Science and Technology, Shanghai 200093, China; qiupeizhen@126.com (P.Q.); lvtaiguo@lcu.edu.cn (T.L.); pai3_14@yeah.net (B.Y.); lianjiqing1990@163.com (J.L.); jingming_usst@163.com (M.J.); 2 Department of Applied Physics, Huzhou University, Huzhou 313000, China; 3 Zhejiang Province Institute of Metrology, Hangzhou 310000, China; beibei210363@126.com

**Keywords:** plasmonic device, multiple beam shaping functionalities, polarization-dependent devices

## Abstract

Realizing multiple beam shaping functionalities in a single plasmonic device is crucial for photonic integration. Both plasmonic Bessel-like beams and bottle beams have potential applications in nanophotonics, particularly in plasmonic based circuits, near field optical trapping, and micro manipulation. Thus, it is very interesting to find new approaches for simultaneous generation of surface plasmon polariton Bessel-like beams and bottle beams in a single photonic device. Two types of polarization-dependent devices, which consist of arrays of spatially distributed sub-wavelength rectangular slits, are designed. The array of slits are specially arranged to construct an X-shaped or an IXI-shaped array, namely X-shaped device and IXI-shaped devices, respectively. Under illumination of circularly polarized light, plasmonic zero-order and first-order Bessel-like beams can be simultaneously generated on both sides of X-shaped devices. Plasmonic Bessel-like beam and bottle beam can be simultaneously generated on both sides of IXI-shaped devices. By changing the handedness of circularly polarized light, for both X-shaped and IXI-shaped devices, the positions of the generated plasmonic beams on either side of device can be dynamically interchanged.

## 1. Introduction

Surface plasmon polaritons (SPPs) are electromagnetic surface waves that propagating along the metal and dielectric interface with field intensity exponentially decaying away from the interface [1]. Owing to SPPs’ unique properties, such as a short wavelength, large local field enhancement, tightly confined in all three dimensions around the metal/dielectric surface [2], SPPs have been utilized in nanoscale optical information transmission and processing. For instance, microscopy [3] and nanoscale lithography [4,5], nano-optical tweezers [6,7,8], as well as surface plasmon circuitry [9].

However, due to the inherent diffraction nature of waves and the ohmic thermal effects in metals, SPPs can only travel a limited distance along the metal surface, with the order of micrometers or nanometers. In order to make full use of the advantages of surface plasmons and overcome the problem of limited propagation distance, recently, in-plane excitation and manipulation of SPP beams have attracted researchers’ attention. Researchers have proposed a series of two-dimensional (2D) optical surface beams with properties of non-diffracting and self-reconstructing, such as generation of plasmonic Bessel-like [10,11,12,13,14,15,16,17,18,19], Airy [20,21], Mathieu and Weber [22], bottle beams [23,24,25,26,27] and arbitrary bending plasmonic light beams [28,29]. Among these nondiffracting SPP beams, plasmonic Bessel-like beam is the most common one, which has a straight trajectory and maintains its transverse profile over a certain distance. Meanwhile, plasmonic bottle beam, which features a single bottle or an array of bottles, presents a bright (or dark) foci surrounded by low (or intense) intensities or oscillating low and high intensity foci. Both plasmonic Bessel-like beam and bottle beam have potential applications in nanophotonics, particularly in plasmonic based near field optical trapping and micro manipulation [2,6,7,8]. In addition, plasmonic Bessel-like beam can also be used for high-resolution fluorescence imaging, high-sensitivity bio-sensing, and near-field on-chip optical communication. Plasmonic bottle beam can also be used to optically sort micro-particles by trapping those with a specific size, and then be applied to fixed-point Raman enhancement and high-resolution Raman spectroscopy imaging.

So far, several plasmonic-based devices have been proposed for generating plasmonic Bessel-like beams and plasmonic bottle beams, separately. An artificially designed 2D micro/nano metallic structure is often used as the coupling structure to convert free-space light into SPP waves with desired wave-fronts. For instance, plasmonic Bessel-like beams were generated by intersecting metallic gratings forming an angle [10,11,12,13,14,15], nonperiodic nanohole array on a metal film [16], a simply metallic grating illuminated by the coupled light with designed phase distribution provided by a spatial light modulator [17], a single triangular dielectric sub-wavelength lens [18], or via surface plasmon excitation with a tightly focused radially polarized beam [19]. Plasmonic bottle beams were generated by two pairs of intersecting grooves [23], three groups of metallic gratings [24], a special binary phase mask [25], a four-slit structure under illumination of multiple-incident Gaussian beams with different phases [26], two slits or one slit structure illuminated by multiple incident lights [27]. Most of the above-mentioned studies utilize gratings (or grooves or slits with length on the order of micrometers) as coupling structures. However, the use of metallic gratings as a plasmonic coupler also has some limitations. Firstly, gratings are polarization dependent plasmonic device. Only the component of incident light that is polarized perpendicularly to the metallic gratings can be coupled into SPP waves. Because of this, transverse magnetic polarized incident light is frequently utilized to illuminate the gratings [30]. Secondly, when the grating structure is illuminated by incident light, two SPP plane waves which propagate in opposite directions are simultaneously excited on both sides of the grating structure. However, only one of the two SPP plane waves is utilized to further generate the target beams (such as plasmonic Bessel-like beam or plasmonic bottle beam). The other one is not utilized and becomes stray light. Thus, the grating structure is not fully exploited. In this paper, this stray light is fully utilized to generate another kind of SPP beam, which can effectively improve the utilization efficiency of the bi-directionally excited SPP waves. Thirdly, it should be noted that most of the proposed grating-based plasmonic devices are just for a single beam shaping functionality. That is, for a fixed device, only one kind of static surface plasmon field distribution is generated. Hence, up to now, plasmonic Bessel-like beam and plasmonic bottle beam are separately generated by using different devices.

Very recently, an increased interest has been focused on designing and incorporating multiple functionalities into a single plasmonic device [31] (which is frequently referred to as metasurface, that is composed of arrays of subwavelength metallic slit units) [32]. In order to achieve multiple functions in a single device, one common method is to change the polarization state or wavelength of incident light. For instance, a polarization-switchable and wavelength-controllable transmissive multiple functional metasurface for flat focusing and controlling the excitation of SPPs at the desired interfaces has been realized [33]. By changing the handedness of circularly polarized light, simultaneous airy beam generation for both SPPs and transmitted wave based on a metasurface has been demonstrated [34]. By changing the polarization states of incident light, SPP Bessel-like beams with different profiles can be selectively generated [35]. Polarization dependent metasurface lenses which can simultaneously focus both SPPs and transmitted wave have been designed [36]. Depending on the helicity of the incident light, two independent functionalities of a hologram and a lens on the same metasurface has been accomplished [37]. A bifunctional metasurface which enables both focusing and anomalous reflection under different polarizations was realized [38]. However, it should be noted that polarization has not yet been utilized to dynamically manipulate plasmonic bottle beam. Furthermore, as far as we know, simultaneous generation of plasmonic Bessel-like beam and plasmonic bottle beam by a single plasmonic device has not yet been reported.

In this work, polarization controllable multi-functional plasmonic devices that allow for simultaneous generation of plasmonic Bessel-like beams and plasmonic bottle beams are designed. The proposed devices are composed of arrays of sub-wavelength rectangular slits etched in a metal film which are designed to construct an X-shaped or an IXI-shaped array. The array of sub-wavelength slits are specially arranged to construct an X-shaped or an IXI-shaped array, namely X-shaped device and IXI-shaped devices, respectively. Under the normal illumination of circularly polarized light, for X-shaped devices, plasmonic zero-order and plasmonic first-order Bessel-like beams can be simultaneously generated on both sides of the device. For IXI-shaped devices, plasmonic Bessel-like beam and plasmonic bottle beam can be simultaneously generated on both sides of the device. By changing the handedness of circularly polarized light, for both X-shaped and IXI-shaped device, the positions of the generated plasmonic beams on either side of the device can be dynamically interchanged. The design scheme of the proposed device provides a new means for constructing plasmonic devices with multiple beam shaping functionalities.

## 2. Results and Discussions

In this section, the design process to realize the proposed plasmonic device is expounded. The working principles of the two types of devices are explained by the polarization selective coupling mechanism of sub-wavelength slits. Simulation results performed with finite difference time domain (FDTD) method are given and discussed.

**Figure 1:**
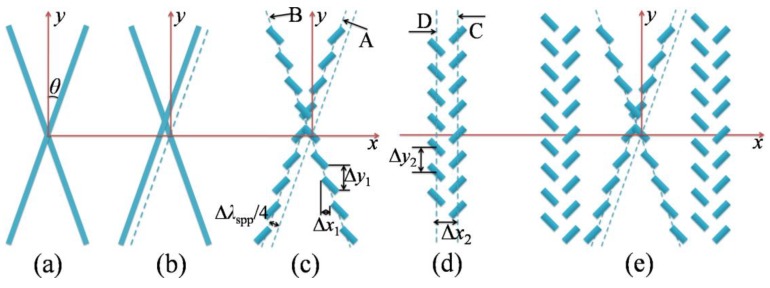
The design flow chart of the proposed plasmonic device. (**a**) A fork-type grating structure. (**b**,**c**) Discrete fork grating into distributed subwavelength slit arrays, to constitute an X-shaped device. (**d**) An I-shaped device consisting of double columns of subwavelength slits with the function of unidirectional launching of SPP waves. (**e**) The designed IXI shaped plasmonic device.

Figure 1 shows the detailed design process to realize the proposed plasmonic device. In order to overcome the limitations of the grating-based coupling structure in controlling SPP Bessel beams and SPP bottle beams, firstly, the fork-type grating structure is discretized into an X-shaped device which is composed of a plurality of sub-wavelength slit units as shown in Figure 1a–c. The sub-wavelength slit arrays are etched in the silver film and can couple the free space light to the bidirectional SPP waves. Secondly, the X-shaped plasmonic device (Figure 1c) is combined with two columns of sub-wavelength slits (Figure 1d) which has unidirectional SPP launching function [39], to constitute an IXI-shaped plasmonic device (Figure 1e). The definitions of geometrical parameters marked in Figure 1 are given. *θ* is defined as the angle between the long axis of the grating and the *y*-axis. Δ*x*_1_ and Δ*y*_1_ are defined as the spacing between adjacent slit units along the *x*-axis and *y*-axis, and Δ*x*_1_ = Δ*y*_1_·tan*θ*. In Figure 1d, Δ*x*_2_ denotes the spacing between two columns of sub-wavelength slits, and Δ*y*_2_ denotes the spacing between adjacent slit units along the *y*-axis. In our paper, high quality quasi plane waves are required to achieve the desired SPP beams (i.e., plasmonic Bessel-like beam or plasmonic bottle beam), because the desired SPP beams are generated by mutual interference of quasi-plane SPP waves excited by single lined slit arrays ‘A’, ‘B’, ‘C’, and ‘D’. It has been demonstrated in [40] that, when the distance between the adjacent nanoslits (such as Δ*y*) is shorter than the effective wavelength of the SPPs (λ_spp_ and λ_spp_ = 613 nm in our paper), a quasi-plane SPP wave can be generated by an array of nanoslits with the same titled angle on the normal illumination of circularly polarized light. A dramatic transition occurs near Δ*y* = λ_spp_*,* which is related to the diffraction limit of the light. Furthermore, such a phenomenon is observed for arbitrary tilted angles [40]. Basing on these discoveries, in our paper, the following structural parameters are given. For the X-shaped plasmonic device *θ* = 10°, Δ*x*_1_ = 0.0353 μm, Δ*y*_1_ = 0.2 μm. The length and width of a single nanoslit unit are 200 nm and 30 nm. For IXI-shaped devices, *θ* = 14°, Δ*x*_1_ = 0.0499 μm, Δ*y*_1_ = 0.2 μm, Δ*x*_2_ = 0.153 μm, Δ*y*_2_ = 0.2 μm. The length and width of a single nanoslit unit are 200 nm and 40 nm. It should be pointed out that before discretizing the fork grating structure, the gratings ‘A’ is shifted by a quarter of the SPP wavelength along the direction perpendicular to grating ‘A’ relative to its original position (Figure 1b). The reason to shift grating ‘A’ will be explained in detail below.

The advantages of discretizing the fork type grating structure into a series of sub-wavelength slit units are as follows. The metallic sub-wavelength slit units interact with the incident light that result in changes in the optical properties (amplitude and phase) of the incident light. It has been found that the control of the phase with metallic sub-wavelength slit units can be related, in some cases, to the fundamental physics associated with the Pancharatnam-Berry (PB) phase [41]. When the polarization state of incident light follows a geodesic triangle on the Poincaré sphere, this geometrical phase appears [42]. The desired PB phase can be obtained by controlling the orientation angle of the slits and the handedness of incident circularly polarized light. The sign of the PB phase depends on the chirality of the incident light. The size of the PB phase depends on the orientation angle (*α*) of each slit unit. Thus, the PB phase approximately follows *φ*(*α*) = σ_±_·[*α*-sgn(*α*)·π/2], where σ_±_ = ±1 represent left-handed circularly polarized (LCP) light and right-handed circularly polarized (RCP) light, respectively [34]. Using the polarization selectivity of the arrays of sub-wavelength slits, the desired phase of the generated plasmonic beam can be set directly through the spatial arrangement of slit arrays [40,43,44].

In the following, the working principle of simultaneous generating mutiple plasmonic beams in a single plasmonic device (i.e., X-shaped plasmonic device and IXI-shaped plasmonic device) will be given.

For the X-shaped plasmonic device, as shown in Figure 1c, when the device is normally illuminated by circularly polarized light, two SPP plane waves can be generated on each side of the device. For instance, on the right side of the device, two SPP plane waves with different propagation directions can be excited. One is generated by single lined slit array ‘A’, and the other is generated by single lined slit array ‘B’. The angles between SPP plane wave propagation directions with respect to the *x*-axis are −*θ* and *θ*, respectively for single lined slit array ‘A’ and single lined slit array ‘B’. These two SPP plane waves will interfere with each other at the angle of 2*θ*, resulting in the generation of corresponding SPP field distribution. Similarly, on the left side of the device, there will be two other SPP plane waves with different propagation directions (±*θ*) separately generated by single lined slit array ‘A’ and single lined slit array ‘B’. Another kind of SPP field distribution will be generated as the result of interference of these two SPP plane waves. In addition, as shown in Figure 1c, the position of the single lined slit array ‘A’ is shifted a distance of a quarter of SPP wavelength relative to its original position. This will lead to an additional π/2 phase shift imposing on the SPP plane wave generated by the single lined slit array ‘A’. The phase difference between the two plane waves that propagating to the right side of the device can be expressed as Δ*φ*_right_ = −λ_spp_/4·k_spp_ + *φ*_B_ − *φ*_A_. Correspondingly, the phase difference between the two plane waves propagating to the left side of the device can be written as Δ*φ*_left_ = λ_spp_/4·k_spp_ + *φ*_B_ *− φ*_A_. Here, *φ*_A_ and *φ*_B_ denote the PB phases induced by the interaction between the incident circularly polarized light and the single lined slit array ‘A’ and ‘B’, respectively. λ_spp_ denotes the wavelength of SPPs. k_spp_ denotes wavenumber of SPPs. The orientation angles (*α*) of the corresponding slit units with respect to *x*-axis in single lined slit array ‘A’ and single lined slit array ‘B’ are *α*_A_ = π/4 and *α*_B_ = −π/4, respectively. According to the principles and demonstration results given in references [10,11,12,13], the plasmonic Bessel-like beams can be generated by mutual interference of two SPP plane waves. Therefore, plasmonic beams with the profiles of zero-order or first-order Bessel-like function can be selectively generated by changing the phase difference between the two SPP plane waves locating on either side of the device.

When LCP light is normally incident on the device, the PB phases are *φ*_A_ = −π/4 and *φ*_B_ = π/4. On right side of the device, Δ*φ*_right_ = 0, which means the two SPP plane waves are in phase and can constructively interfere along the positive *x*-axis. Thus, the plasmonic zero-order Bessel-like beam can be generated [10,11]. On left side of the device, Δ*φ*_left_ = π, which means the two SPP plane waves are out of phase and can destructively interfere along the negative *x*-axis. Thus, the plasmonic first-order Bessel-like beam can be generated [12,13].

In brief, for LCP light, plasmonic zero-order Bessel-like beam and plasmonic first-order Bessel-like beam can be simultaneously generated by using a single device. The plasmonic zero-order Bessel-like beam is generated on the right side of the device. The plasmonic first-order Bessel-like beam is generated on the left side of the device.

When RCP light is normally incident on the device, the additional PB phases are *φ*_A_ = π/4 and *φ*_B_ *=* −π/4. On right side of the device, Δ*φ*_right_ = −π. On left side of the device, Δ*φ*_left_ = 0. It can be seen that the plasmonic zero-order and first-order Bessel-like beams can be also simultaneously generated by using a single plasmonic device. However, for RCP light, different from the case of LCP light, the plasmonic first-order Bessel-like beam is generated on the right side of the device. The plasmonic zero-order Bessel-like beam is generated on the left side of the device. Therefore, the positions of the two types of beams are dependent on the handedness of circularly polarized light and can be dynamically interchanged by changing the handedness of circularly polarized light.

**Figure 2:**
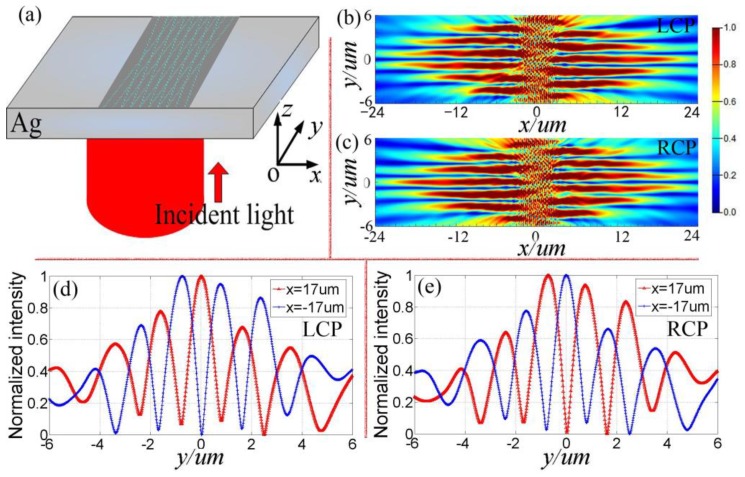
Numerical verifications on the feasibility of simultaneously generating plasmonic zero-order and first-order Bessel-like beams by using a single device. (**a**) Schematic diagram of the optical configuration for X-shaped plasmonic devices. FDTD simulated SPP field intensity for the X-shaped device illuminated by (**b**) LCP light and (**c**) RCP light. As a comparison, the normalized intensity values along y-axis at *x* = ±17 μm in (**b**,**c**) are extracted and plotted in (**d**,**e**), respectively. (**d**) for LCP light and (**e**) for RCP light.

The correctness of the above working principle for X-shaped plasmonic device is numerically demonstrated by using commercial software Lumerical FDTD Solutions. Figure 2 shows the numerical verification results on the feasibility of simultaneously generating plasmonic zero-order and first-order Bessel-like beams by using a single X-shaped device. Figure 2a shows a schematic diagram of the optical configuration for X-shaped devices. In order to effectively improve the coupling efficiency, the cross-section of the device is increased by placing eight pairs of X-shaped slit arrays, which are spaced 600 nm apart. The device is back normally illuminated with a Gaussian beam with the waist radius of 20 μm. The wavelength of the incident light is λ_o_ = 633 nm. The dielectric permittivity of the silver is ε = −15.9317 + i1.07633 at 633 nm, and the corresponding SPP wavelength is λ_spp_ = 613 nm. The thickness of the silver film is 120 nm. The length and width of a single slit unit are 200 nm and 30 nm, respectively. *θ* = 10°, Δ*x*_1_ = 0.0353 μm, Δ*y*_1_ = 0.2 μm. Figure 2b,c show the generated SPP field intensity distributions at the plane 80 nm above the device for LCP light and RCP light, respectively. From Figure 2b,c, it can be seen that, under the illumination with circularly polarized light, two different types of plasmonic beams which separately locate at both sides of the device can be simultaneously generated by a single plasmonic device. The normalized intensity values along *y*-axis at *x* = ±17 μm for SPP intensity field generated by LCP light and RCP light are extracted and shown in Figure 2d,e, respectively. For both LCP light and RCP light, it can be seen that plasmonic zero-order Bessel-like beam and first-order Bessel-like beam respectively locate on the right side and left side of the device. However, the corresponding positions of the generated two types of plasmonic Bessel-like beams are different and depended on the handedness of the incident circularly polarized light. This can be understood by checking the above derived phase distributions (Δ*φ*) of generated SPP waves on both sides of the device for LCP and RCP light. In [35], an axicon-shaped slit array is designed to dynamically manipulate Bessel-like SPP beams (plasmonic zero-order Bessel-like beam and first-order Bessel-like beam) by modulating the polarization of the incident light. However, only the SPP beams on one side of the device are utilized. Under the illumination of incident light with a certain polarization state, only one type of Bessel-like plasmonic beams is generated. Compared with [35], in this paper, with a certain polarization state, two types of plasmonic Bessel-like beams are simultaneously generated, and are located on both sides of the device respectively.

Next, the working principle of the IXI-shaped plasmonic device is introduced. The IXI-shaped device (Figure 1e) is a combination of an X-shaped device and an I-shaped device. The I-shaped device is composed of two parallel columns of slit units, namely column ‘C’ and column ‘D’ shown in Figure 1d. The I-shaped device can realize the function of polarization-controlled tunable directional launching of SPP waves. This structure was firstly proposed by Lin, and the detailed analysis of mechanism for achieving directional launching of SPP waves can be found in the literature [39]. The two parallel columns are spaced λ_spp_/4 apart. The orientation angles of slit units for column ‘C’ and column ‘D’ are *α*_C_ = π/4, *α*_D_ = −π/4, respectively. Under the illumination of circularly polarized light, on each side of the device, two SPP plane waves were generated by column ‘C’ and column ‘D’. The phase differences between the two plane waves propagating to the right side of the device can be expressed as Δ*φ*_right_ = λ_spp_/4·k_spp_ + *φ*_D_ − *φ*_C_. The phase differences between the two plane waves propagating to the left side of the device can be expressed as Δ*φ*_left_ = − λ_spp_/4·k_spp_ + *φ*_D_ − *φ*_C_. The corresponding interference field intensity distributions on both sides of the I-shaped device can be expressed as, *I*_right_ ∝ *E*_D_^2^ + *E*_C_^2^ + 2·*E*_D_·*E*_C_·cos(Δ*φ*_right_), and *I*_left_ ∝ *E*_D_^2^ + *E*_C_^2^ + 2·*E*_D_·*E*_C_·cos(Δ*φ*_left_). *E*_D_ and *E*_C_ denote the amplitude of the two SPP plane waves. Therefore, by controlling the magnitude of the phase differences (i.e., Δ*φ*_right_ and Δ*φ*_left_), constructive interference or destructive interference occurs between the corresponding two SPP plane waves on either side of the device, thereby realizing the function of SPP unidirectional launching.

**Figure 3:**
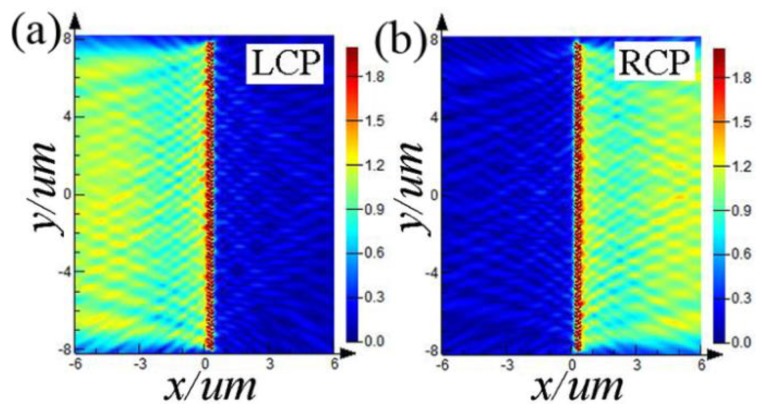
Numerical verifications on the feasibility of polarization controlled tunable directional launching of SPP waves by an I-shaped device shown in Figure 1d under illumination with (**a**) LCP light and (**b**) RCP light.

Figure 3 shows the numerically FDTD simulation results of the directional launching SPP waves by changing the handedness of circularly polarized light. When the incident light is LCP light, the additional PB phases are *φ*_C_ = −π/4, *φ*_D_ = π/4. On right side of the device, Δ*φ*_right_ = π. On left side of the device, Δ*φ*_left_ = 0. Thus, *I*_right_ = 0, I_left_ ≠ 0, which indicates that the launched SPP waves propagate perpendicularly away toward left side of the device, as shown in Figure 3a. When the incident light is RCP light, the additional PB phases are *φ*_C_ = π/4, *φ*_D_ = −π/4. On right side of the device, Δ*φ*_right_ = 0. On left side of the device, Δ*φ*_left_ = −*π*. Thus, *I*_right_ ≠ 0, *I*_left_ = 0, which indicates that the launched SPP waves propagate perpendicularly away toward right side of the device, as shown in Figure 3b. Thus, the observed SPP intensity field distributions shown in Figure 3 are in good agreement with the above theoretical analysis.

Based on the above analysis of the working principles for the X-shaped device and the I-shaped device, it can be inferred that the IXI-shaped device, which is composed of the two, has the following characteristics. Under the illumination with LCP light, three SPP plane waves are generated on the left side of the device. As have been demonstrated by [24], the plasmonic bottle beam can be generated by the mutual interference of three SPP plane waves. However, on the right side of the device, only two SPP plane waves are generated, and the phase difference between these two plane waves is zero and therefore the plasmonic zero-order Bessel-like beam is generated. Similarly, under the illumination with RCP light, three SPP plane waves are generated on the right side of the device, and only two SPP plane waves with phase difference Δ*φ*_right_ = 0 are generated on the left side of the device. Therefore, for LCP light, plasmonic bottle beam can be generated on the right side of the device, and plasmonic zero-order Bessel-like beam can be generated on the left side of the device.

**Figure 4:**
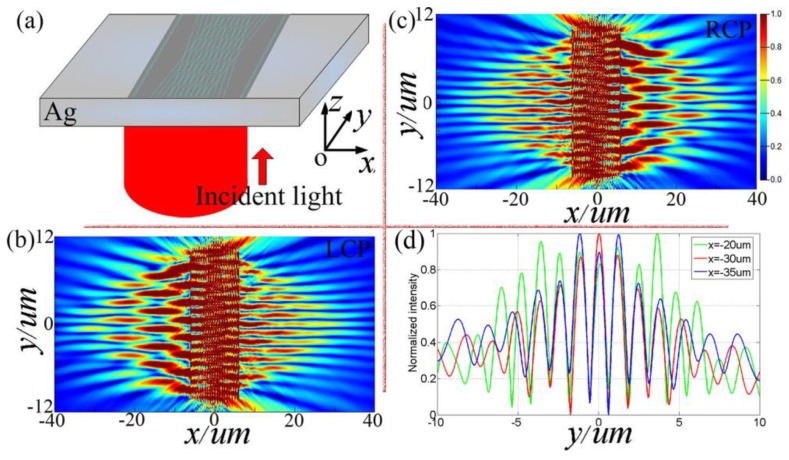
Numerical verifications on the feasibility of simultaneously generating plasmonic Bessel-like beam and plasmonic bottle beam by a single device. (**a**) Schematic diagram of the optical configuration for designed IXI-shaped devices. Numerical simulation results of SPP field intensity distributions generated by LCP light (**b**) and RCP light (**c**). As a comparison, the normalized intensity profiles along *y*-axis at *x* = 20 μm, 30 μm, 35 μm for RCP light shown in (**c**) are extracted and plotted in (**d**).

Figure 4 shows the numerical verification results on the feasibility of simultaneously generating plasmonic Bessel-like beam and plasmonic bottle beam by using a single IXI-shaped device using FDTD method. Figure 4a gives a schematic diagram of the optical configuration for the IXI-shaped device. The cross-section of the device is increased by placing eleven pairs of X-shaped slit arrays, which are spaced 600 nm apart. For IXI-shaped device, *θ* = 14°, Δ*x*_1_ = 0.0499 μm, Δ*y*_1_ = 0.2 μm, Δ*x*_2_ = 0.153 μm, Δ*y*_2_ = 0.2 μm. The length and width of a single slit unit are 200 nm and 40 nm, respectively. The parameters of incident light and silver layer are the same as those shown in Figure 2a. Figure 4b shows the generated SPP field intensity distribution at the plane 30 nm above the device for LCP light. Figure 4c shows the corresponding SPP field intensity distribution for RCP light. It can be seen that under the illumination of circularly polarized light, the device can simultaneously generate plasmonic bottle beams and plasmonic zero-order Bessel-like beams. However, for LCP light, the generated plasmonic bottle beam is located on the left side of the device, and the generated plasmonic Bessel-like beam is located on the right side of the device. In contrast, for RCP light, the generated bottle beam is located on the right side of the device, and the generated Bessel-like beam is located on the left side of the device.

The normalized SPP field intensity distributions along *y*-axis at *x* = 20 μm, 30 μm, 35 μm in Figure 4c are extracted and plotted in Figure 4d. The average spatial periods calculated through the curves shown in Figure 4c are about 1.2038 μm, 1.2172 μm, 1.2648 μm (corresponding to *x* = 20 μm, 30 μm, 35 μm). Meanwhile, the average spatial period of the fringes formed by the interference of two SPP plane waves along the *y*-axis can be expressed as: T = λ_spp_/2/sin(*θ*) = 0.613/2/sin(14°) = 1.2669 μm. The corresponding absolute errors are 0.0631 μm, 0.0497 μm, 0.0021 μm. The errors are much smaller than the wavelength of SPPs, which indicate the generated Bessel-like beam has diffraction invariance within a certain distance.

## 3. Conclusions

Realizing multiple beam shaping functionalities by using a single plasmonic device is crucial for photonic integration. It is very interesting to find new approaches to control the shapes of multiple SPP wavefronts in a single photonic device.

In this work, a kind of wavefont-controllable and position-switchable plasmonic device is designed, which can realize simultaneous excitation for plasmonic Bessel-like beams and bottle beams in a single device, depending on the polarization state of incident light. Two types of devices, namely X-shaped device and IXI-shaped device, are presented. The proposed devices consist of arrays of spatially distributed sub-wavelength rectangular slits. Under the illumination of circularly polarized light, plasmonic zero-order Bessel-like beam and first-order Bessel-like beam are simultaneously generated on the surface of the X-shaped device (separately located on both sides of the device), and plasmonic Bessel-like beam and plasmonic bottle beam are simultaneously generated on the surface of the IXI-shaped device (separately located on both sides of the device). By changing the handedness of circularly polarized light, the corresponding positions of the generated beams can be also dynamically interchanged.

The design process to realize the proposed plasmonic device is illustrated. The working principles of the two types of devices are explained by the polarization selective coupling mechanism of sub-wavelength slits. Under the normal illumination of incident light with different polarization states, the phase effects of a single slit unit on the incident light are different. Thus, the desired phase difference can be obtained by spatially arranging the sub-wavelength slits and changing the handedness of circularly polarized light. Simulation results performed with FDTD method are given and are consistent with the theoretical predictions.

The innovations of this paper are as follows. (1) The designed device makes full use of SPP waves excited on both sides of the device. Whether it is X-shaped device or IXI-shaped device, two different types of SPP beams can be generated simultaneously on both sides of the device. The utilization efficiency of the bi-directionally excited SPP waves can be effectively improved. Moreover, the corresponding positions of the generated SPP beams can be dynamically switched by changing the handedness of the circularly polarized light; (2) A new means for constructing surface plasmon device with multiple beam shaping functionalities is provided. Firstly, some established beam shaping plasmonic devices are discretized into sub-wavelength slit unit arrays, and secondly combined with the plasmonic device with directional emission function, to finally form polarization-dependent multi-beam shaping plasmonic devices.

In summary, the design scheme of the proposed provides a new means for constructing plasmonic devices with multiple beam shaping functionalities. The presented device suggests promising applications in polarization-controlled integrated plasmonic circuits and surface optical trapping and micro manipulation.
